# Blood creatinine and urea nitrogen at ICU admission and the risk of in-hospital death and 1-year mortality in patients with intracranial hemorrhage

**DOI:** 10.3389/fcvm.2022.967614

**Published:** 2022-11-10

**Authors:** Hai Luo, Xuanyong Yang, Kang Chen, Shihai Lan, Gang Liao, Jiang Xu

**Affiliations:** ^1^Department of Neurosurgery, The First Affiliated Hospital, Nanchang University, Nanchang, China; ^2^Institute of Medicine, Nanchang University, Nanchang, China

**Keywords:** intracerebral hemorrhage, creatinine, urea nitrogen, hospital death, 1-year mortality

## Abstract

**Background:**

The relationship between renal function and clinical outcomes in patients with intracranial hemorrhage is controversial.

**Aims:**

We investigated the associations of blood creatinine and urea nitrogen levels with hospital death and 1-year mortality in patients with intracranial hemorrhage treated in the intensive care unit (ICU).

**Methods:**

A total of 2,682 patients with intracranial hemorrhage were included from the Medical Information Mart for Intensive Care III (MIMIC-III) database. Clinical variables, including admission creatinine, urea nitrogen, type of intracranial hemorrhage, underlying diseases and other blood biochemistry parameters, were collected. Multivariable correction analysis was conducted of the relationships between blood creatinine and urea nitrogen levels on admission with hospital death and 1-year mortality in the included patients with intracranial hemorrhage. Smooth curve and subgroup analyses were also performed for these associations.

**Results:**

A total of 2,682 patients had their blood creatinine and urea nitrogen levels measured within the first 24 h after ICU admission, with median values of 0.80 and 15.00 mg/dL, respectively. We observed steeply linear relationships between creatinine and urea nitrogen levels and the risk of in-hospital death and 1-year mortality, but the risk of in-hospital mortality and 1-year mortality increased little or only slowly above creatinine levels > 1.9 mg/dL or urea nitrogen > 29 mg/d (the inflection points). Consistently, conditional logistic regression analysis suggested that these inflection points had significant modification effects on the associations between blood creatinine levels, as well as blood urea nitrogen, and the risk of in-hospital death (interaction value < 0.001) and 1-year mortality (interaction value < 0.001).

**Conclusion:**

Our results supported the hypothesis that elevated blood creatinine and urea nitrogen levels on admission are associated with an increased risk of in-hospital death and 1-year mortality in patients with intracranial hemorrhage. Interestingly, these independent relationships existed only for lower levels of serum creatinine (<1.9 mg/dL) and uric acid (<29 mg/dL).

## Introduction

Some known chronic diseases, such as hypertension, cardiac disease and diabetes, and unhealthy lifestyles, including smoking, drinking and a high-fat diet, have been found to contribute to a high stroke risk (1). Renal dysfunction has been considered a critical indicator of adverse clinical outcomes, including stroke (2, 3).

Blood creatinine and urea nitrogen, as the key indices used to evaluate renal function, play an important role in assessing the severity of the status of intensive care unit (ICU) patients, especially after cardiovascular events (4). For instance, chronic kidney disease (CKD) was found to be associated with a higher rate of adverse clinical outcomes and mortality risk, and elevated blood creatinine levels had good value in predicting short-term and long-term mortality in patients with hemorrhagic and ischemic stroke (5, 6). Previous studies also reported that admission dehydration status was independently related to an increased risk of 30-day mortality (7). Conversely, one previous study unexpectedly found that the admission estimated glomerular filtration rate (eGFR) in patients with intracranial hemorrhage, calculated using serum creatinine according to the Modification of Diet in Renal Disease (MDRD) equation, was not associated with in-hospital mortality (8). Other clinical studies have reported that admission dehydration and a blood urea-to-creatinine ratio > 80 significantly contribute to a reduced risk of in-hospital mortality in older patients with acute intracranial hemorrhage (9). The inconsistent findings among studies might be caused by populations with various age groups, different sample sizes, different stroke types, different concomitant diseases, different measurement times of serum parameters and other confounding variables. For example, malignant tumors may increase short-term mortality in patients with cardiovascular events (10). Stoke patients with intracranial hemorrhage in different parts of the brain have obviously different prognoses (11). More importantly, follow-up studies with large samples on the association between renal function and mortality risk in patients with intracranial hemorrhage are scarce.

Given the current research evidence, we collected data from a total of 2,682 ICU patients from the Medical Information Mart for Intensive Care III (MIMIC-III) database. The MIMIC-III database included enough patients with intracranial hemorrhage, and data for clinical variables, including comorbidities and blood biochemical parameters, were collected. The present study investigated and further verified the associations of blood creatinine and urea nitrogen levels on admission with intracranial hemorrhage-associated in-hospital death and 1-year mortality in ICU patients with intracranial hemorrhage.

## Materials and methods

### Study population

The MIMIC III (v1.4) database was used for the present study. As a single-center critical care database, the MIMIC-III is publicly available and was approved for use by the Institutional Review Boards of the Massachusetts Institute of Technology (MIT, Cambridge, MA, USA) and Beth Israel Deaconess Insurance Center (BIDMC, Boston, MA, USA). Written informed consent was obtained from each patient based on the Declaration of Helsinki guidelines.

The database contains detailed clinical information, including clinical variables such as demographic characteristics, vital signs, vital status and laboratory tests, on 46,520 patients admitted to various ICUs of the BIDMC from 2001 to 2012 in Boston, Massachusetts (12). ICU nurses recorded the physiological data obtained from bedside monitors hourly. International Classification of Diseases and Ninth Revision (ICD-9) codes were used for disease diagnosis in the present study. The use of data from this database is not considered as human subject research, and there is no requirement for patient consent due to the anonymized health information. However, users (scientific researchers) need to pass an examination to register for use of the database and must be approved by the administration staff of the MIMIC-III database. The users are allowed to use the database to extract data for study analysis only after passing the “Protecting Human Research Participants” course available on the National Institutes of Health (NIH) website.

### Subject selection

ICU patients included in the MIMIC-III database who experienced intracranial hemorrhage were enrolled in our study. Intracranial hemorrhage was caused by various etiologies, including head trauma, spontaneous intracranial hemorrhage and unknown causes, according to ICD-9 codes. First, to ensure the accuracy of the review, we excluded all patients aged < 18 years and >90 years or those who had important missing variables due to common missing data in the MIMIC-III database. Then, all patients for whom initial creatinine and urea nitrogen measurements were completed within the first 24 h after ICU admission were included. In addition, we only analyzed the first ICU stay for patients who were admitted to the ICU more than once. We used the first recorded value of clinical characteristics in the ICU.

### Variable extraction

The initial exposure variables were whether these ICU patients experienced “intracranial hemorrhage”. Structured query language (SQL) was used. The first recorded value of clinical characteristics at baseline during the ICU stay were obtained, including age, sex, length of ICU stay, insurance status, ICU type, type of intracranial hemorrhage, head trauma, comorbidities, blood test parameters and medications.

Blood test parameters, including creatinine, urea nitrogen, sodium, potassium, phosphate, magnesium, total calcium, chloride, glucose, partial thromboplastin time (PTT), prothrombin time (PT), international normalized ratio (INR), red blood cell count, platelet count, white blood cell count and bicarbonate, were tested within the first 24 h after ICU admission. If these blood variables were recorded more than once in the first 24 h, we used the first recorded value.

The type of intracranial hemorrhage mainly included “intracerebral,” “extradural,” “subdural,” “unspecified intracranial,” “subarachnoid,” and “multiple.” Head trauma was defined as “yes” or “no”. ICU types included cardiology care unit (CCU), cardiovascular surgery rehabilitation unit (CSRU), insurance intensive care unit (MICU), surgical intensive care unit (SICU) and trauma/surgical intensive care unit (TSICU). Medications mainly included vasopressin, nitrate esters, β receptor blockers, benzodiazepines, statins and potassium. Additionally, data on comorbidities were obtained, including heart failure, hypertension, myocardial infarction, cerebral infarction, diabetes, chronic kidney disease and malignant tumor, according to the ICD-9 codes recorded in the MIMIC-III database.

### Clinical outcomes

For the purpose of this study, the clinical endpoints were classified as in-hospital death and 1-year morality. In-hospital death was defined as “the patient died during hospitalization”. The definition of 1-year mortality was “the patient died within 1 year of ICU admission”.

### Statistical analysis

The normality of the data was examined for continuous variables by the Kolmogorov–Smirnov (KS) test. Values are expressed as medians [interquartile ranges (IQRs)] due to non-normal distributions of all continuous variables in our study. All categorical variables are expressed as total numbers and percentages. Comparisons between two groups (patients with in-hospital death vs. those without in-hospital death; patients with 1-year mortality vs. those without 1-year mortality) were made using the Mann–Whitney U test for continuous variables and the chi-square test for categorical variables.

First, multivariate logistic regression models were used to characterize the associations between blood creatinine and urea nitrogen levels at baseline and the clinical outcomes (risk of in-hospital death and 1-year mortality). Other confounding factors (baseline variables) that were clinically relevant to the clinical outcomes, including age, sex, hypertension, heart failure, myocardial infarction, diabetes and cerebral infarction, were entered into a multivariate model as covariates. Then subgroup analysis using “type of intracranial hemorrhage” and “head trauma” as grouping variables was performed for these associations. Because malignancy and insurance status are important causes of all-cause death in elderly individuals, we also used “malignant tumors” and “insurance” as covariates in the sensitivity analysis.

Additionally, we performed a smooth curve analysis for relationships between blood creatinine and urea nitrogen levels and the risk of in-hospital death and 1-year mortality. A smoothed curve can fully demonstrate the non-linear associations of two variables. Stratified analysis was further performed on the associations of blood creatinine and urea nitrogen levels with the risk of in-hospital death and 1-year mortality through the inflection points (blood creatinine = 1.9 mg/dL and blood urea nitrogen = 29 mg/dL) of the smooth curves. In the stratification analysis, conditional logistic regression analysis was used, and the modification effect of the inflection points on the associations between blood creatinine and urea nitrogen levels with the risk of in-hospital death and 1-year mortality was assessed. All statistical analyses were performed by using Stata 13.0 and EmpowerStats 3.0. A *P*-value < 0.05 was defined as statistically significant.

## Results

### Clinical characteristics at baseline

**Figure 1:**
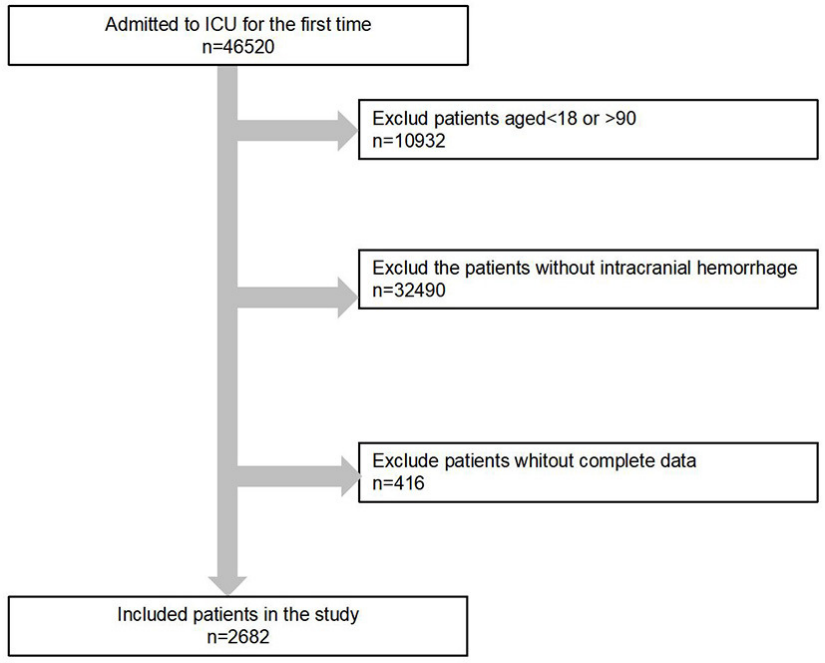
The flow chart of patient inclusion in the study.

After reviewing the data of 46,520 patients, a total of 2,682 ICU patients with intracranial hemorrhage were enrolled in the present study, as shown in the flow chart of the included study subjects (Figure 1). Among our study cohort, the median age of all patients was 66.5 years, and 1,057 (51.2%) of them were female. Blood creatinine and urea nitrogen levels were measured for all 2,682 patients within the first 24 h after ICU admission, with median values of 0.80 and 15.00 mg/dL, respectively. The median ICU stay was 2.92 (1.54–7.12) days. All patients were classified into two groups according to survival state (alive or dead). Importantly, patients in the non-surviving group were significantly older, had a longer ICU stay, had higher blood creatinine and urea nitrogen levels and had a higher rate of comorbidities (heart failure, myocardial infarction, cerebral infarction and malignant tumor) than those in the surviving group. Their other clinical characteristics at baseline are described in detail in Table 1.

**Table 1:** Clinical characteristics of ICU patients with intracranial hemorrhage at baseline.

**Variable**	**All = 2,682**	**In-hospital death**			**1-year mortality**		
		**Alive = 2,157**	**Dead = 525**	***p***	**Alive = 1,831**	**Dead = 851**	***p***
Age (year)	66.5 (52.5–78.8)	64.8 (51.4–78.1)	73.4 (59.1–80.8)	< 0.001	62.8 (50.0–75.8)	75.1 (61.4–82.3)	< 0.001
Gender (male), *n* (%)	1,507 (56.19)	1,221 (56.61)	286 (54.48)	0.378	1,037 (56.64)	470 (55.23)	0.494
Days of ICU stay	2.92 (1.54–7.12)	2.83 (1.50–7.33)	3.67 (1.75–6.88)	0.092	2.75 (1.42–7.12)	3.38 (1.75–7.10)	0.002
**Insurance**							
Government, *n* (%)	87 (3.24)	82 (3.80)	5 (0.95)	< 0.001	81 (4.42)	6 (0.71)	< 0.001
Medicaid, *n* (%)	221 (8.24)	186 (8.62)	35 (6.67)		170 (9.28)	51 (5.99)	
Medicare, *n* (%)	1,341 (50.00)	1,012 (46.92)	329 (62.67)		768 (41.94)	573 (67.33)	
Private, *n* (%)	969 (36.13)	834 (38.66)	135 (25.71)		771 (42.11)	198 (23.27)	
Self pay, *n* (%)	64 (2.39)	43 (1.99)	21 (4.00)		41 (2.24)	23 (2.70)	
**First care unit**							
CCU, *n* (%)	74 (2.76)	48 (2.23)	18 (3.43)	0.041	43 (2.35)	23 (2.70)	< 0.001
CSRU, *n* (%)	64 (2.39)	66 (3.06)	19 (3.62)		55 (3.00)	30 (3.53)	
MICU, *n* (%)	375 (13.98)	273 (12.66)	88 (16.76)		213 (11.63)	148 (17.39)	
SICU, *n* (%)	1,381 (51.49)	1,074 (49.79)	244 (46.48)		913 (49.86)	405 (47.59)	
TSICU, *n* (%)	788 (29.38)	696 (32.27)	156 (29.71)		607 (33.15)	245 (28.79)	
**Comorbidities**							
Heart failure, *n* (%)	285 (10.63)	209 (9.69)	76 (14.48)	0.001	156 (8.52)	129 (15.16)	< 0.001
Hypertension, *n* (%)	1,358 (50.63)	1,086 (50.35)	272 (51.81)	0.548	915 (49.97)	443 (52.06)	0.315
Miocardial infarction, *n* (%)	28 (1.04)	17 (0.79)	11 (2.10)	0.008	13 (0.71)	15 (1.76)	0.013
Cerebral infarction, *n* (%)	185 (6.90)	138 (6.40)	47 (8.95)	0.038	107 (5.84)	78 (9.17)	0.002
Diabetes, *n* (%)	31 (1.16)	26 (1.21)	5 (0.95)	0.627	23 (1.26)	8 (0.94)	0.476
Malignant tumor, *n* (%)	128 (4.77)	104 (4.82)	24 (4.57)	0.810	53 (2.89)	75 (8.81)	< 0.001
Chronic kidney disease, *n* (%)	107 (3.99)	79 (3.66)	28 (5.33)	0.079	56 (3.06)	51 (5.99)	< 0.001
**Blood test parameters**							
Sodium (mEq/L)	139.00 (137.00–141.00)	139.00 (137.00–141.00)	139.00 (137.00–142.00)	0.154	139.00 (137.00–141.00)	139.00 (137.00–142.00)	0.898
Potassium (mEq/L)	3.90 (3.50–4.20)	3.90 (3.50–4.20)	3.80 (3.50–4.30)	0.728	3.90 (3.50–4.20)	3.90 (3.50–4.30)	0.837
Phosphate (mg/dL)	3.20 (2.70–3.70)	3.20 (2.80–3.70)	3.00 (2.50–3.80)	0.001	3.20 (2.80–3.70)	3.10 (2.50–3.75)	0.004
Magnesium (mg/dL)	1.90 (1.70–2.00)	1.90 (1.70–2.00)	1.80 (1.60–2.00)	0.514	1.90 (1.70–2.00)	1.80 (1.60–2.00)	0.556
Total calcium (mg/dL)	8.60 (8.10–9.00)	8.60 (8.20–9.00)	8.50 (8.00–9.00)	0.014	8.60 (8.20–9.00)	8.50 (8.00–9.00)	0.045
Chloride (mEq/L)	105.00 (102.00–108.00)	105.00 (102.00–108.00)	105.00 (101.00–109.00)	0.969	105.00 (102.00–108.00)	104.00 (101.00–108.00)	0.278
Glucose (mg/dL)	134.00 (112.00–166.00)	130.00 (110.00–160.00)	154.00 (127.00–198.00)	< 0.001	129.00 (109.00–157.00)	147.00 (123.00–189.50)	< 0.001
PTT (sec)	26.60 (24.20–29.70)	26.50 (24.40–29.40)	26.70 (23.90–31.30)	0.118	26.30 (24.20–29.20)	27.00 (24.20–31.20)	< 0.001
PT (sec)	13.30 (12.60–14.30)	13.20 (12.60–14.10)	13.60 (12.70–15.30)	< 0.001	13.20 (12.50–14.00)	13.60 (12.70–15.10)	< 0.001
INR PT	1.20 (1.10–1.30)	1.10 (1.10–1.30)	1.20 (1.10–1.40)	< 0.001	1.10 (1.10–1.20)	1.20 (1.10–1.40)	< 0.001
Red blood cell count (m/uL)	3.88 (3.42–4.32)	3.91 (3.45–4.32)	3.78 (3.31–4.33)	0.028	3.95 (3.49–4.34)	3.75 (3.27–4.25)	< 0.001
Platelet count (K/uL)	214.00 (166.00–268.00)	215.00 (168.00–268.00)	211.00 (151.00–271.00)	0.073	218.00 (171.50–269.50)	204.00 (149.50–264.50)	< 0.001
White blood cell count (K/uL)	10.55 (7.90–13.70)	10.20 (7.70–13.00)	12.50 (9.20–16.00)	< 0.001	10.10 (7.70–12.90)	11.90 (8.50–15.10)	< 0.001
Bicarbonate (mEq/L)	24.00 (22.00–26.00)	24.00 (22.00–26.00)	23.00 (21.00–26.00)	< 0.001	24.00 (22.00–26.00)	24.00 (21.00–26.00)	< 0.001
Creatinine (mg/dL)	0.80 (0.70–1.10)	0.80 (0.70–1.00)	0.90 (0.70–1.30)	< 0.001	0.80 (0.70–1.00)	0.90 (0.70–1.20)	< 0.001
Urea nitrogen (mg/dL)	15.00 (11.00–20.00)	15.00 (11.00–20.00)	17.00 (13.00–26.00)	< 0.001	14.00 (10.00–19.00)	17.00 (13.00–25.00)	< 0.001
**Medications**							
Vasopressin, *n* (%)	105 (3.91)	44 (2.04)	61 (11.62)	< 0.001	31 (1.69)	74 (8.70)	< 0.001
Nitrate esters, *n* (%)	112 (4.18)	96 (4.45)	16 (3.05)	0.150	73 (3.99)	39 (4.58)	0.473
β Receptor blocker, *n* (%)	1,369 (51.04)	1,116 (51.74)	253 (48.19)	0.145	928 (50.68)	441 (51.82)	0.583
Benzodiazepine, *n* (%)	186 (6.94)	140 (6.49)	46 (8.76)	0.066	117 (6.39)	69 (8.11)	0.103
Statins, *n* (%)	277 (10.33)	242 (11.22)	35 (6.67)	0.002	189 (10.32)	88 (10.34)	0.988
Potassium, *n* (%)	357 (13.31)	288 (13.35)	69 (13.14)	0.899	241 (13.16)	116 (13.63)	0.739
**Type of intracranial hemorrhage**							
Intracerebral, *n* (%)	971 (36.20)	727 (33.70)	244 (46.48)	< 0.001	573 (31.29)	398 (46.77)	< 0.001
Extradural, *n* (%)	15 (0.56)	11 (0.51)	4 (0.76)		10 (0.55)	5 (0.59)	
Subdural, *n* (%)	625 (23.30)	546 (25.31)	79 (15.05)		453 (24.74)	172 (20.21)	
Unspecified intracranial, *n* (%)	212 (7.90)	170 (7.88)	42 (8.00)		153 (8.36)	59 (6.93)	
Subarachnoid, *n* (%)	756 (28.19)	618 (28.65)	138 (26.29)		566 (30.91)	190 (22.33)	
Multiple, *n* (%)	103 (3.84)	85 (3.94)	18 (3.43)		76 (4.15)	27 (3.17)	
**Head trauma**, *n* (%)	1,005 (37.47)	862 (39.96)	143 (27.24)	< 0.001	754 (41.18)	251 (29.49)	< 0.001

ICU, intensive care unit; CCU, cardiology care unit; CSRU, cardiovascular surgery rehabilitation unit; MICU, medical intensive care unit; SICU, surgical intensive care unit; TSICU, trauma/surgical intensive care unit; PTT, partial thromboplastin time; PT, prothrombin time; INR, international normalized ratio.

### Elevated blood creatinine and urea nitrogen levels were related to an increased risk of in-hospital death and 1-year mortality

The multivariate logistic regression analysis demonstrated significantly adverse associations of elevated blood creatinine levels with an increased risk of in-hospital death [unadjusted odds ratio (OR) = 1.14, 95% CI 1.05–1.22, *P* = 0.001] and 1-year mortality (unadjusted OR = 1.28, 95% CI 1.17–1.40, *P* < 0.001), as shown in Table 2. Similar associations existed for urea nitrogen, with a higher risk of in-hospital death (unadjusted OR = 1.02, 95% CI 1.02–1.03, *P* < 0.001) and 1-year mortality (unadjusted OR = 1.03, 95% CI 1.03–1.04, *P* < 0.001). These results remained consistent after adjusting for confounding factors, including age, sex, hypertension, heart failure, myocardial infarction, diabetes and cerebral infarction. Furthermore, we used “malignant tumor” and “insurance” as covariates in the sensitivity analysis. We observed that this independent relationship changed only slightly (Table 3).

**Table 2:** Corrected logistic regression analysis for associations of blood creatinine and urea nitrogen with hospital mortality and 1-year mortality in ICU patients with intracranial hemorrhage.

**Variable**	**In-hospital death**		**1-year mortality**	
	**OR (95% CI)**	***P*** **-value**	**OR (95% CI)**	***P*** **-value**
**Unadjusted**				
Creatinine (mg/dL)	1.14 (1.05, 1.22)	0.001	1.28 (1.17, 1.40)	< 0.001
Urea Nitrogen (mg/dL)	1.02 (1.02, 1.03)	< 0.001	1.03 (1.03, 1.04)	< 0.001
**Plus age**				
Creatinine (mg/dL)	1.13 (1.04, 1.21)	0.002	1.24 (1.14, 1.35)	< 0.001
Urea nitrogen (mg/dL)	1.02 (1.01, 1.02)	< 0.001	1.02 (1.02, 1.03)	< 0.001
**Plus gender**				
Creatinine (mg/dL)	1.13 (1.05, 1.22)	0.002	1.24 (1.14, 1.35)	< 0.001
Urea nitrogen (mg/dL)	1.02 (1.01, 1.02)	< 0.001	1.02 (1.02, 1.03)	< 0.001
**Plus hypertension**				
Creatinine (mg/dL)	1.12 (1.04, 1.21)	0.003	1.22 (1.12, 1.33)	< 0.001
Urea nitrogen (mg/dL)	1.02 (1.01, 1.02)	< 0.001	1.02 (1.02, 1.03)	< 0.001
**Plus heart failure**				
Creatinine (mg/dL)	1.11 (1.03, 1.20)	0.005	1.21 (1.11, 1.31)	< 0.001
Urea nitrogen (mg/dL)	1.02 (1.01, 1.02)	< 0.001	1.02 (1.01, 1.03)	< 0.001
**Plus miocardial infarction**				
Creatinine (mg/dL)	1.11 (1.03, 1.20)	0.005	1.21 (1.11, 1.31)	< 0.001
Urea nitrogen (mg/dL)	1.02 (1.01, 1.02)	< 0.001	1.02 (1.01, 1.03)	< 0.001
**Plus diabetes**				
Creatinine (mg/dL)	1.12 (1.03, 1.20)	0.004	1.21 (1.11, 1.32)	< 0.001
Urea nitrogen (mg/dL)	1.02 (1.01, 1.02)	< 0.001	1.02 (1.01, 1.03)	< 0.001
**Plus cerebral infarction**				
Creatinine (mg/dL)	1.12 (1.03, 1.20)	0.005	1.22 (1.12, 1.33)	< 0.001
Urea nitrogen (mg/dL)	1.02 (1.01, 1.02)	< 0.001	1.02 (1.02, 1.03)	< 0.001

ICU, intensive care unit.

**Table 3:** Sensitivity analysis for associations of blood creatinine and urea nitrogen with hospital mortality and 1-year mortality in ICU patients with intracranial hemorrhage.

**Variable**	**In-hospital death**		**1-year mortality**	
	**OR (95% CI)**	***P*** **-value**	**OR (95% CI)**	***P*** **-value**
**Model 1 (Plus malignant)**				
Creatinine (mg/dL)	1.12 (1.03, 1.20)	0.005	1.22 (1.12, 1.33)	< 0.001
Urea nitrogen (mg/dL)	1.02 (1.01, 1.02)	< 0.001	1.02 (1.01, 1.03)	< 0.001
**Model 2 (Plus insurance)**				
Creatinine (mg/dL)	1.10 (1.02, 1.19)	0.016	1.20 (1.10, 1.31)	< 0.001
Urea nitrogen (mg/dL)	1.02 (1.01, 1.02)	< 0.001	1.02 (1.01, 1.03)	< 0.001

**Model 1:** Adjusted for age, gender, hypertension, heart failure, miocardial infarction, diabetes, cerebral infarction and malignan.

**Model 2:** Adjusted for age, gender, hypertension, heart failure, miocardial infarction, diabetes, cerebral infarction, malignant and insurance.

ICU, intensive care unit.

We also used subgroup analysis to evaluate patients with different types of intracranial hemorrhage and head trauma for these associations. Our results showed that only in patients with subarachnoid hemorrhage increased blood creatinine levels were associated with an elevated risk of in-hospital death [adjusted odds ratio (OR) = 1.68, 95% CI 1.14–2.47, *P* = 0.009] and 1-year mortality (adjusted OR = 1.65, 95% CI 1.10–2.47, *P* = 0.015), as shown in Table 4. However, a significant association did not exist among patients with intracerebral, subdural and unspecified intracranial hemorrhage. This indicates that different types of intracranial hemorrhage have different risks of death. Additionally, we also observed that elevated blood creatinine and urea nitrogen levels contributed to a higher risk of in-hospital death and 1-year mortality among patients without head trauma but not among those with head trauma (Table 5). However, head trauma did not have a significant modifying effect on the relationship.

**Table 4:** Subgroup analysis by “type of intracranial hemorrhage” for associations of blood creatinine and urea nitrogen with hospital mortality and 1-year mortality in ICU patients in cerebral hemorrhage.

**Type of cerebral hemorrhage**	**Variable**	**In-hospital death**		**1-year mortality**	
		**OR (95% CI)**	***P*** **-value**	**OR (95% CI)**	***P*** **-value**
Intracerebral	Creatinine (mg/dL)	1.09 (0.99, 1.21)	0.093	1.27 (1.09, 1.48)	0.002
	Urea nitrogen (mg/dL)	1.01 (1.00, 1.02)	0.003	1.02 (1.01, 1.03)	< 0.001
Extradural	Creatinine (mg/dL)	–	–	–	–
	Urea nitrogen (mg/dL)	–	–	–	–
Subdural	Creatinine (mg/dL)	1.00 (0.85, 1.18)	0.994	1.10 (0.97, 1.24)	0.148
	Urea nitrogen (mg/dL)	1.02 (1.01, 1.03)	0.003	1.02 (1.01, 1.03)	< 0.001
Unspecified intracrania	Creatinine (mg/dL)	1.13 (0.69, 1.86)	0.629	0.92 (0.55, 1.54)	0.755
	Urea nitrogen (mg/dL)	1.01 (0.98, 1.04)	0.431	1.00 (0.97, 1.03)	0.844
Subarachnoid	Creatinine (mg/dL)	1.68 (1.14, 2.47)	0.009	1.65 (1.10, 2.47)	0.015
	Urea nitrogen (mg/dL)	1.04 (1.02, 1.06)	< 0.001	1.03 (1.01, 1.06)	0.002
Multiple	Creatinine (mg/dL)	3.79 (0.57, 25.31)	0.169	3.92 (0.58, 26.73)	0.163
	Urea nitrogen (mg/dL)	1.02 (0.96, 1.08)	0.521	1.04 (0.97, 1.12)	0.283

Adjusted for age, gender, hypertension, heart failure, miocardial infarction, diabetes, cerebral infarction, malignant and insurance.

ICU, intensive care unit.

**Table 5:** Subgroup analysis by “head trauma” for associations of blood creatinine and urea nitrogen with hospital mortality and 1-year mortality in ICU patients with intracranial hemorrhage.

**Variable**	**Head trauma**	**In-hospital death**			**1-year mortality**		
		**OR (95% CI)**	***P*** **-value**	^*^ *P*	**OR (95% CI)**	***P*** **-value**	^*^ *P*
Creatinine (mg/dL)	No	1.11 (1.01, 1.22)	0.027	0.317	1.18 (1.07, 1.31)	0.002	0.927
	Yes	1.00 (0.83, 1.21)	0.987		1.17 (1.00, 1.37)	0.053	
Urea nitrogen (mg/dL)	No	1.02 (1.01, 1.03)	< 0.001	0.504	1.02 (1.01, 1.03)	< 0.001	0.266
	Yes	1.01 (1.00, 1.03)	0.063		1.01 (1.00, 1.03)	0.035	

Adjusted for age, gender, hypertension, heart failure, miocardial infarction, diabetes, cerebral infarction, malignant and insurance.

* *P* for interaction value.

### Stratified analysis using the “inflection point” of the smooth curve for associations between blood creatinine and urea nitrogen levels and the risk of in-hospital death and 1-year mortality

**Figure 2:**
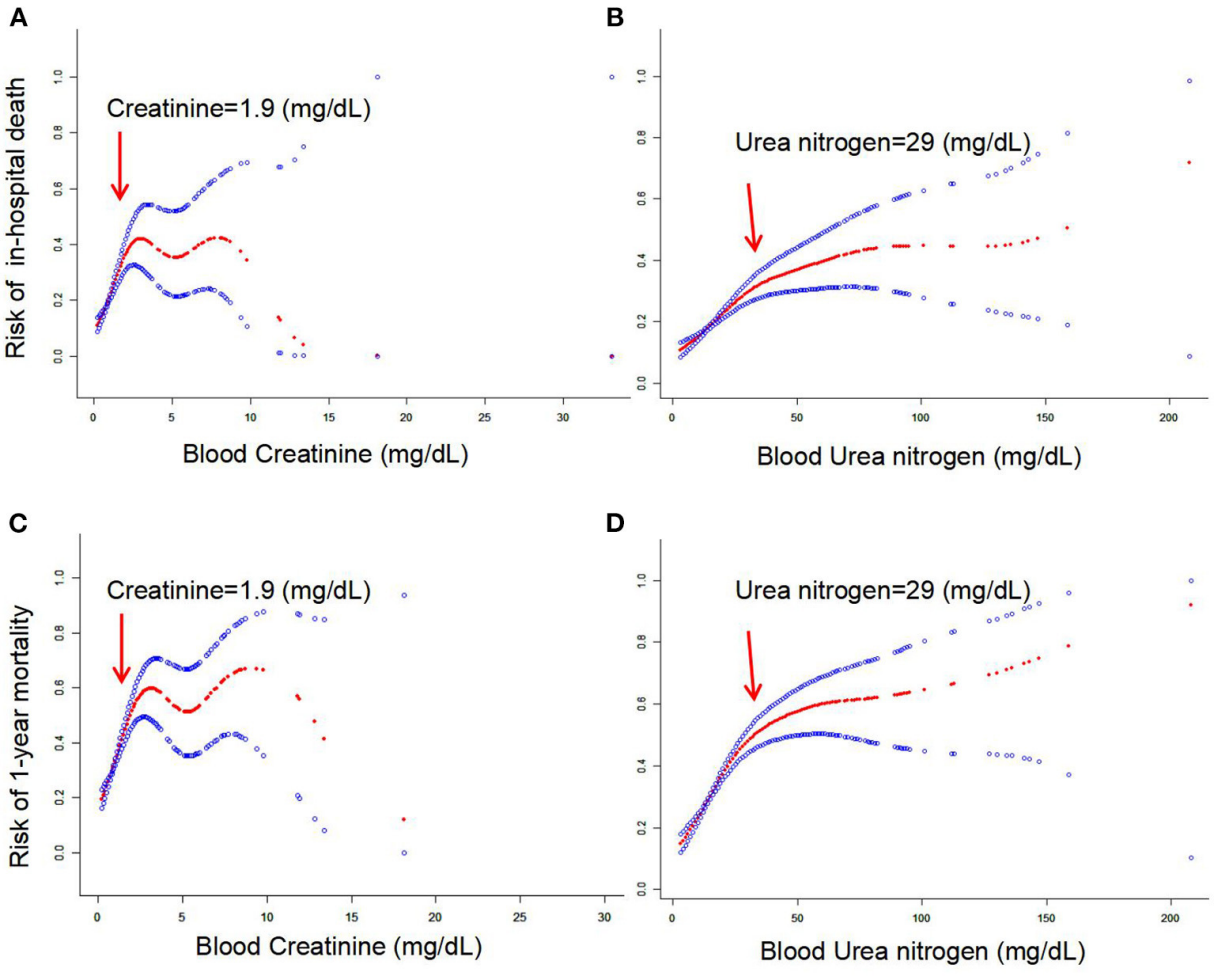
**(A–D)** Smoothed curves of the associations between blood creatinine and urea nitrogen and mortality risk in patients with intracranial hemorrhage.

To further analyze the relationships between blood creatinine and urea nitrogen and clinical outcomes, we used a smooth curve to find potential “inflection points” of the relationships. Surprisingly, the smooth curve revealed a gradual upward trend in the association between blood creatinine and the risk of in-hospital death and 1-year mortality (Figures 2A,C; inflection point of creatinine = 1.9 mg/dL). Similar results existed for urea nitrogen (Figures 2B,D; inflection point of urea nitrogen = 29 mg/dL). When the blood creatinine value was < 1.9 mg/dL, we observed a steeply linear relationship between the creatinine level and the risk of in-hospital death and 1-year mortality, while the risk of in-hospital death and 1-year mortality increased only slightly or very slowly when the creatinine level exceeded 1.9 mg/dL. These results suggested that increased creatinine levels mainly strongly contributed to a higher risk of in-hospital death and that 1-year mortality increased only when the creatinine values were below 1.9 mg/dL. Similarly, we found that blood urea nitrogen levels were significantly related to the risk of in-hospital death and 1-year mortality when the blood urea nitrogen value was < 29 mg/dL, whereas the risk of in-hospital death and 1-year mortality increased very little or only very slowly after the creatinine level exceeded 29 mg/d.

According to the inflection point (blood creatinine = 1.9 mg/dL and blood urea nitrogen = 29 mg/dL) of the smooth curve, stratified analysis was further used to analyze the associations of blood creatinine and urea nitrogen levels with the risk of clinical outcomes, as shown in Table 6. Consistently, conditional logistic regression analysis suggested that the inflection points had significant modification effects on the associations between blood creatinine levels, as well as blood urea nitrogen, and the risk of in-hospital death (interaction value < 0.001) and 1-year mortality (interaction value < 0.001).

**Table 6:** Interaction analysis by “inflection point” for associations of blood creatinine and urea nitrogen with hospital mortality and 1-year mortality in ICU patients with intracranial hemorrhage.

**Variable**	**In-hospital death**			**1-year mortality**		
	**OR (95% CI)**	***P*** **-value**	^*^ *P*	**OR (95% CI)**	***P*** **-value**	^*^ *P*
**Crude**						
Creatinine < 1.9 (mg/dL)	2.50 (1.80, 3.45)	< 0.001	< 0.001	2.48 (1.86, 3.30)	< 0.001	< 0.001
Creatinine ≥ 1.9 (mg/dL)	0.90 (0.79, 1.03)	0.116		1.01 (0.92, 1.11)	0.814	
Urea nitrogen < 29 (mg/dL)	1.06 (1.03, 1.08)	< 0.001	< 0.001	1.07 (1.05, 1.09)	< 0.001	< 0.001
Urea nitrogen ≥ 29 (mg/dL)	1.01 (1.00, 1.02)	0.187		1.01 (1.00, 1.02)	0.077	
**Plus age**						
Creatinine < 1.9 (mg/dL)	2.00 (1.43, 2.79)	< 0.001	< 0.001	1.71 (1.27, 2.30)	< 0.001	0.001
Creatinine ≥ 1.9 (mg/dL)	0.89 (0.78, 1.02)	0.087		1.01 (0.92, 1.11)	0.804	
Urea nitrogen < 29 (mg/dL)	1.03 (1.01, 1.05)	0.004	0.022	1.03 (1.01, 1.05)	0.001	0.042
Urea nitrogen ≥ 29 (mg/dL)	1.00 (1.00, 1.01)	0.344		1.01 (1.00, 1.02)	0.068	
**Plus gender**						
Creatinine < 1.9 (mg/dL)	2.27 (1.60, 3.22)	< 0.001	< 0.001	1.78 (1.30, 2.44)	< 0.001	0.001
Creatinine ≥ 1.9 (mg/dL)	0.90 (0.79, 1.02)	0.108		1.02 (0.92, 1.12)	0.756	
Urea nitrogen < 29 (mg/dL)	1.03 (1.01, 1.06)	0.002	0.018	1.03 (1.01, 1.05)	0.001	0.045
Urea nitrogen ≥ 29 (mg/dL)	1.00 (1.00, 1.01)	0.350		1.01 (1.00, 1.02)	0.070	
**Plus hypertension**						
Creatinine < 1.9 (mg/dL)	2.24 (1.57, 3.17)	< 0.001	< 0.001	1.75 (1.27, 2.40)	< 0.001	0.002
Creatinine ≥ 1.9 (mg/dL)	0.92 (0.81, 1.04)	0.168		1.02 (0.92, 1.13)	0.699	
Urea nitrogen < 29 (mg/dL)	1.04 (1.01, 1.06)	0.002	0.015	1.03 (1.01, 1.05)	< 0.001	0.029
Urea nitrogen ≥ 29 (mg/dL)	1.00 (0.99, 1.01)	0.400		1.01 (1.00, 1.02)	0.104	
**Plus heart failure**						
Creatinine < 1.9 (mg/dL)	2.20 (1.54, 3.13)	< 0.001	< 0.001	1.70 (1.24, 2.34)	0.001	0.003
Creatinine ≥ 1.9 (mg/dL)	0.92 (0.81, 1.04)	0.170		1.02 (0.92, 1.12)	0.703	
Urea nitrogen < 29 (mg/dL)	1.04 (1.01, 1.06)	0.002	0.017	1.03 (1.01, 1.05)	0.001	0.035
Urea nitrogen ≥ 29 (mg/dL)	1.00 (0.99, 1.01)	0.434		1.01 (1.00, 1.02)	0.111	
**Plus miocardial infarction**						
Creatinine < 1.9 (mg/dL)	2.20 (1.55, 3.14)	< 0.001	< 0.001	1.70 (1.24, 2.34)	0.001	0.003
Creatinine ≥ 1.9 (mg/dL)	0.92 (0.82, 1.04)	0.204		1.02 (0.93, 1.13)	0.635	
Urea nitrogen < 29 (mg/dL)	1.04 (1.01, 1.06)	0.002	0.015	1.03 (1.01, 1.05)	0.001	0.031
Urea nitrogen ≥ 29 (mg/dL)	1.00 (0.99, 1.01)	0.433		1.01 (1.00, 1.02)	0.111	
**Plus diabetes**						
Creatinine < 1.9 (mg/dL)	2.22 (1.56, 3.16)	< 0.001	< 0.001	1.72 (1.25, 2.36)	0.001	0.002
Creatinine ≥ 1.9 (mg/dL)	0.92 (0.81, 1.04)	0.190		1.03 (0.93, 1.13)	0.624	
Urea nitrogen < 29 (mg/dL)	1.04 (1.01, 1.06)	0.001	0.013	1.03 (1.01, 1.05)	0.001	0.027
Urea nitrogen ≥ 29 (mg/dL)	1.00 (0.99, 1.01)	0.438		1.01 (1.00, 1.02)	0.118	
**Plus cerebral infarction**						
Creatinine < 1.9 (mg/dL)	2.20 (1.54, 3.14)	< 0.001	< 0.001	1.70 (1.23, 2.34)	0.001	0.003
Creatinine ≥ 1.9 (mg/dL)	0.91 (0.81, 1.02)	0.110		1.02 (0.91, 1.13)	0.740	
Urea nitrogen < 29 (mg/dL)	1.04 (1.01, 1.06)	0.002	0.016	1.03 (1.01, 1.05)	0.001	0.034
Urea nitrogen ≥ 29 (mg/dL)	1.00 (0.99, 1.01)	0.439		1.01 (1.00, 1.02)	0.125	
**Plus malignant**						
Creatinine < 1.9 (mg/dL)	2.20 (1.54, 3.14)	< 0.001	< 0.001	1.71 (1.24, 2.35)	0.001	0.003
Creatinine ≥ 1.9 (mg/dL)	0.90 (0.80, 1.02)	0.089		1.01 (0.89, 1.13)	0.916	
Urea nitrogen < 29 (mg/dL)	1.04 (1.01, 1.06)	0.002	0.014	1.03 (1.01, 1.05)	0.001	0.036
Urea nitrogen ≥ 29 (mg/dL)	1.00 (0.99, 1.01)	0.434		1.01 (1.00, 1.02)	0.104	
**Plus insurance**						
Creatinine < 1.9 (mg/dL)	2.16 (1.51, 3.09)	< 0.001	< 0.001	1.68 (1.21, 2.32)	0.002	0.003
Creatinine ≥ 1.9 (mg/dL)	0.89 (0.78, 1.01)	0.067		0.99 (0.87, 1.14)	0.930	
Urea nitrogen < 29 (mg/dL)	1.04 (1.01, 1.06)	0.001	0.026	1.03 (1.01, 1.06)	< 0.001	0.061
Urea nitrogen ≥ 29 (mg/dL)	1.01 (1.00, 1.02)	0.204		1.01 (1.00, 1.02)	0.032	

Inflection point for creatinine < 1.9 mg/dL (*n* = 2,538) and creatinined ≥ 1.9 mg/dL (*n* = 144); Inflection point for urea nitrogen < 29 mg/dL (*n* = 2,362) and Urea nitrogen ≥ 29 mg/dL (*n* = 320).

* *P*: interaction value.

ICU, intensive care unit.

## Discussion

Globally, stroke is an important cause of disability and death, and acute cerebral hemorrhage accounts for ~26% of all strokes (13, 14). The Kidney Early Evaluation Program (KEEP) showed that CKD patients are at a higher risk of both myocardial infarction and stroke when their eGFR is < 60 mL/min/1.73 m^2^ (15). There is also a study reporting a graded association between a reduced eGFR and the risk of adverse cardiovascular outcomes, including stroke (16). However, the relationship between renal function and mortality risk in ICU patients with intracranial hemorrhage has not been studied.

We analyzed 2,682 patients with intracranial hemorrhage from the MIMIC-III database. Consistently, we observed adverse associations between blood creatinine and urea nitrogen levels and the risk of in-hospital death and 1-year mortality in these ICU patients after adjusting for all identified confounding factors. Importantly, we identified new inflection points in which elevated blood creatinine and urea nitrogen levels were associated with an increased risk of in-hospital death and 1-year mortality: a blood creatinine value < 1.9 mg/dL and a blood urea nitrogen value < 29 mg/dL. Beyond these levels, the mortality risk in these patients with intracranial hemorrhage did not increase significantly.

Renal dysfunction has been found to be an independent factor for mortality risk in various populations, including patients with heart failure, patients with myocardial infarction and the general population (17–19). However, previous evidence on the association between renal dysfunction and mortality risk in acute stroke has some heterogeneity. MacWalter et al. observed that patients with increased serum creatinine (1.35 mg/dL) levels at admission had a higher risk of mortality in a cohort of 2,042 patients with stroke followed up for 7 years in Scotland (20). Yeh et al. observed that a reduced eGFR (<60 mL/min/1.73 m^2^) was related to a poor prognosis within 6 months among patients with atherosclerosis stroke (21). Furthermore, Hao et al. found that a low eGFR on admission was a valuable indicator for predicting 1-year mortality in patients with acute intracranial hemorrhage but not in patients with ischemic stroke (22). In contrast, Kumia et al. found that proteinuria, but not a reduced eGFR, as evaluated by the MDRD equation, was significantly associated with an increased risk of poor functional outcomes and mortality after acute ischemic stroke (23). Another study suggested that a low eGFR level on admission, calculated by the MDRD equation, did not contribute to an increased risk of in-hospital mortality in intracranial hemorrhage patients (7). The heterogeneity of these conclusions may derive from the different study populations selected, the different stroke types included, the different methods used to evaluate renal function and the various confounding factors taken into account.

Consistent with some previous studies (20–22), the results of our study suggested that reduced blood creatinine and urea nitrogen levels were associated with a significantly higher risk of either in-hospital death or 1-year mortality in ICU patients with intracranial hemorrhage only when their blood creatinine value was <1.9 mg/dL or their blood urea nitrogen value was <29 mg/dL. The mortality risk in these patients with intracranial hemorrhage did not increase significantly as these measures increased. This confirmed the results of previous smaller studies and provided new and important information that an acute increase in blood creatinine and urea nitrogen levels within the normal range of the general population contributes to a significantly increased risk of hospitalization and long-term mortality.

Cancer and insurance status are important factors for long-term mortality in elderly patients (24–26). In our study, all included individuals were elderly patients, and 128 (4.77%) patients had malignant tumors. In addition to controlling for traditional risk factors such as age, sex and cardiovascular diseases that can affect the long-term survival rate of elderly individuals, our sensitivity analysis demonstrated that malignant tumor and insurance status did not significantly affect the independent association of creatinine and urea nitrogen with mortality risk among patients with intracranial hemorrhage, which was inconsistent with previous studies. Moreover, the findings of this study suggested that increased blood creatinine levels were associated with an elevated risk of in-hospital death and 1-year mortality only in patients with subarachnoid hemorrhage. A significant association did not exist for patients with intracerebral, subdural or unspecified intracranial hemorrhage. This is consistent with the clinicopathological condition that different types of intracranial hemorrhage have different mortality risks.

This study has several notable strengths. (1) Our study used intracranial hemorrhage data from the MIMIC-III database, which contains information from a large number of ICU patients with intracranial hemorrhage. This retrospective cohort study design could estimate the real-world prognosis of intracranial hemorrhage patients. (2) We observed a positive association between blood creatinine and urea nitrogen levels within 24 h of admission and a higher risk of in-hospital death and 1-year mortality in patients with intracranial hemorrhage only when their blood creatinine value was < 1.9 mg/dL or their blood urea nitrogen value was < 29 mg/dL. (3) Demographic characteristics, concomitant cardiovascular disease and malignant tumors were controlled in multivariate analysis, which eliminated the influence of relevant confounding factors. The present study also has several limitations. First, we did not use any information on accurate eGFR levels, which can reflect renal function well. Second, we only used the first value of the blood creatinine level measured on admission. We could not distinguish chronic renal dysfunction from acute renal dysfunction, potentially skewing the results. Third, 416 patients with intracranial hemorrhage were excluded due to missing information (Figure 1). Whether their data might affect the results is uncertain.

In conclusion, our results suggested that elevated blood creatinine and urea nitrogen levels contributed to an increased risk of in-hospital death and 1-year mortality in ICU patients with intracranial hemorrhage only when the blood creatinine value was <1.9 mg/dL or the blood urea nitrogen value was <29 mg/dL.

## Data availability statement

The raw data supporting the conclusions of this article will be made available by the authors, without undue reservation.

## Ethics statement

The studies involving human participants were reviewed and approved by Review Boards of the Massachusetts Institute of Technology (MIT, Cambridge, MA, USA) and Beth Israel Deaconess Insurance Center (BIDMC, Boston, MA, USA), and written informed consent was obtained from each patient based on the Declaration of Helsinki guidelines.

## Author contributions

HL and JX completed the data analysis and first draft. XY, KC, SL, and GL completed surveillance and data duplication. All authors contributed to the article and approved the submitted version.

## Conflict of interest

The authors declare that the research was conducted in the absence of any commercial or financial relationships that could be construed as a potential conflict of interest.

## Publisher's note

All claims expressed in this article are solely those of the authors and do not necessarily represent those of their affiliated organizations, or those of the publisher, the editors and the reviewers. Any product that may be evaluated in this article, or claim that may be made by its manufacturer, is not guaranteed or endorsed by the publisher.
